# The dynamics of benzene on Cu(111): a combined helium spin echo and dispersion-corrected DFT study into the diffusion of physisorbed aromatics on metal surfaces

**DOI:** 10.1039/c7fd00095b

**Published:** 2017-04-27

**Authors:** M. Sacchi, P. Singh, D. M. Chisnall, D. J. Ward, A. P. Jardine, W. Allison, J. Ellis, H. Hedgeland

**Affiliations:** a Department of Chemistry , University of Surrey , Guildford , GU2 7XH , UK . Email: m.sacchi@surrey.ac.uk; b The Perse School , Cambridge , CB2 8QF , UK; c Cavendish Laboratory , University of Cambridge , JJ Thomson Avenue , Cambridge , CB3 0HE , UK; d School of Physical Sciences , The Open University , Walton Hall , Milton Keynes , MK7 6AA , UK

## Abstract

We use helium spin-echo spectroscopy (HeSE) to investigate the dynamics of the diffusion of benzene adsorbed on Cu(111). The results of these measurements show that benzene moves on the surface through an activated jump-diffusion process between the adsorption sites on a Bravais lattice. Density Functional Theory (DFT) calculations with van der Waals (vdW) corrections help us understand that the molecule diffuses by jumping through non-degenerate hollow sites. The results of the calculations shed light on the nature of the binding interaction between this prototypical aromatic molecule and the metallic surface. The highly accurate HeSE experimental data provide a quantitatively stringent benchmark for the vdW correction schemes applied to the DFT calculations and we compare the performances of several dispersion interaction schemes.

## 1. Introduction

The self-assembly of aromatic molecules and polymers on the surface of semiconductors and metals is a key component in the design and production of organo-electronics and photovoltaic devices, as well as being central in corrosion protection and coating technologies. The self-assembly process is characterised by three main factors: the surface-molecule binding, the intermolecular interactions between adsorbed precursors, and the dynamic behaviour (*i.e.*, surface diffusion, rotational entropy and conformational mobility) of the adsorbate. Among these factors, the surface dynamics of the adsorbed precursor are by far the most difficult to study because the complexity of the molecular motion, once it has been activated, and the timescale then involved (around 10^–8^ to 10^–12^ s) mean that commonly employed microscopy techniques are unsuited to the task.^1^ In this study we investigate the adsorption and diffusion of benzene on Cu(111). Benzene is the most fundamental building block in Polycyclic Aromatic Hydrocarbons (PAHs) and forms a part of several of the π-conjugated molecules and polymers employed in organic electronics, such as PTCDA, PTCDI, rubrene, polyphenylene and derivatives.^2^–^5^ The ability to characterise and predict the self-assembly properties of this large class of molecules intrinsically depends on our understanding of the interactions and dynamic behaviour of this iconic six-membered aromatic ring when confined on the surface of single crystals. In previous works we have explored the dynamics of the cyclopentadienyl anion (Cp), pyrrole and thiophene adsorbed on the same Cu(111) surface.^6^–^9^ The combination of state of the art helium spin-echo spectroscopy (HeSE) and density functional theory calculations has provided insight into the role of charge transfer and ionic binding in aromatic adsorption,^8^,^9^ and the contribution of rotational,^6^ vibrational^7^ and other external degrees of freedom^10^ to molecular transport on surfaces.

Notwithstanding the central role of benzene adsorption in surface and interface chemistry, the number of low-coverage, atomically resolved experimental studies of the adsorption of this aromatic molecule on copper and other coinage metal surfaces is relatively limited.^11^–^20^ Early STM and DFT studies by Komeda *et al.*^20^ and by Lorente *et al.*^19^ show that benzene adsorbs in a flat orientation on the Cu(100) surface, with the hollow sites being the preferred adsorption sites.^19^ On Cu(111) the molecule adsorbs up to a single monolayer coverage with the aromatic ring parallel to the surface^17^,^18^ and is able to diffuse freely and form stable islands near the steps at a temperature of 77 K.^17^,^21^ The long series of previous computational studies of benzene adsorption on Cu(111), employing both standard DFT generalised gradient approximation (GGA) exchange–correlation (XC) functionals and dispersion-corrected XC functionals, are motivated by the general lack of agreement on some fundamental results, such as the preferred adsorption site, the adsorption energies and the molecule-surface distance at the equilibrium.^12^ The previous experimental and theoretical studies generally focused on the properties of the C_6_H_6_–Cu(111) system in equilibrium at a given surface temperature and coverage. The dynamical aspects of the self-assembly process, in particular the surface mobility, have not been quantitatively explored. The main reason for the lack of information on the barriers for surface diffusion, as well as the friction coefficients, resides in the difficulty in determining the minimum energy adsorption site and, particularly, in measuring with accuracy the diffusion rates of the molecule. For instance, in the case of thiophene on Cu(111),^8^ three different degrees of freedom – molecular translation, rotation and vertical motion – were individually characterized by DFT and HeSE, giving accurate, quantitative insight into the atomic-scale motion of a heteroaromatic on a surface. In this work we present new theoretical and experimental insights into the adsorption and diffusion of benzene on Cu(111), a paradigmatic surface science system for benchmarking weak dispersion interactions in 2D.

## 2. Methods

### 2.1 Theoretical method

**Fig. 1:**
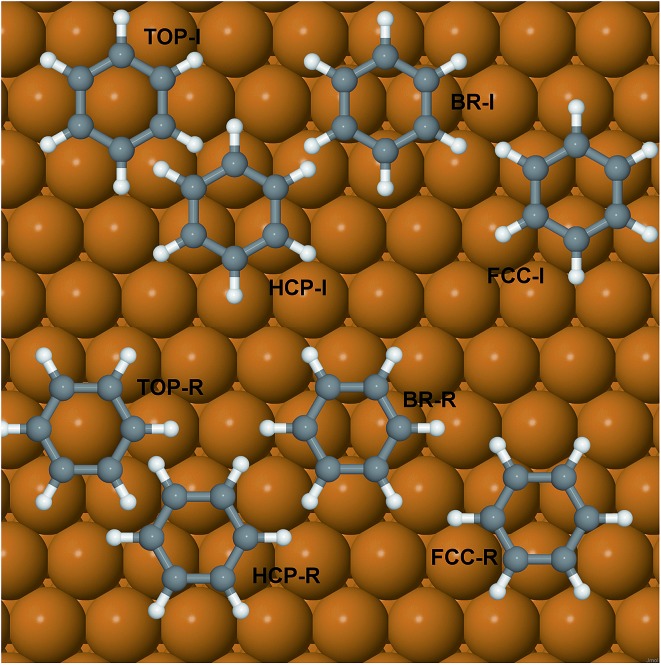
Schematic representation of benzene adsorbed on high symmetry adsorption sites in the two angular orientations (0 and 30 degrees with respect to the <110> direction) considered in this work. In our notation, *I* = 0 degrees rotation and *R* = 30 degrees rotation.

The potential energy surface of benzene on Cu(111) has been mapped by performing a series of first-principles DFT calculations for adsorption on high-symmetry sites: top, bridge and hollow (fcc and hcp). Since the experiments were performed in a low-coverage regime, only the in-plane rotational degree of freedom (the rotation around the central C_6_ molecular axis) was considered and the geometry of the benzene was optimised at the two different initial angular orientations (see Fig. 1), 0 and 30 degrees (with respect to the <110> direction). In the adsorption of aromatics on noble and coinage metal surfaces, the molecule-surface binding is dominated by the long-range dispersion forces, *i.e.*, van der Waals (vdW) forces. It is well-known that DFT, in its classic local density approximation (LDA) or generalised gradient approximation (GGA), is local (or semi-local) in nature; therefore a major effort has been undertaken in the last decade or so to overcome this restriction, by applying correction schemes or *ad hoc* modifications to popular functionals such as PBE or B88. A review of the general strengths and weaknesses of the various approaches is beyond the scope of this paper, but one can draw the case that, as for “non-dispersion corrected” DFT, a classic Ladder^22^ or more modern Stairway^23^ of chemical accuracy *versus* computational cost exists. The right method for a specific system needs to be selected by balancing the desired level of accuracy and overall cost of the calculations. For the present study we have employed first and second step methods,^23^ following the classification of Klimeš and Michaelides, and in order of increasing accuracy these are: DFT-D2 (G06) by Grimme and co-authors,^24^ Ortmann–Bechstedt–Schmidt (OBS) in ref. 25, Tkatchenko–Scheffler (TS),^26^ and the Self-Consistent Tkatchenko–Scheffler (TSSCS) correction^27^ schemes. The exchange–correlation functional of choice is the Perdew–Burke–Ernzerhof (PBE)^28^ functional with the G06, TS, TSSCS corrections and the Perdew–Wang’91 (PW91)^29^ functional for the OBS correction. The PW91 XC-functional is very similar in accuracy to the PBE functional. It has been employed for testing the OBS scheme because, in the original formulation and benchmarking, the functional used was PW91, therefore the C_6_ coefficients available are more compatible with PW91 than with PBE. Given the relatively large dimensions of the (2√3 × 2√3) surface unit cell needed to simulate the experimental conditions, we note the extremely good performance of the TS and related schemes in combination with the GGA exchange–correlation functional, both in terms of the cost and chemical accuracy.

The remaining details of the calculations and convergence criteria have been previously reported and discussed in several recent reports,^6^–^9^ but we summarise here the most important computational parameters. The surface has been modelled as a seven-layer slab, with a vacuum layer of ∼20 Å vertically separating the periodically repeated supercells. The cut-off energy of the plane wave basis set was fixed at 11 Hartrees, while the Brillouin zone was sampled with a 4 × 4 × 1 *k*-point Monkhorst–Pack^30^ grid. The bottom three layers were kept fixed in the bulk crystal positions while the top four atomic layers were allowed to relax during structural optimisation.

### 2.2 Experimental method

Helium spin-echo spectroscopy uses a beam of neutral, nuclear spin-polarised helium-3 atoms to measure the time-dependent correlation of surface adsorbates. Magnetic fields are used to divide the helium beam into spin-dependent wavepackets, which arrive at the surface with a known temporal separation. The interference of the scattered wavepackets results in a measurable polarisation of the beam which varies with the separation time of the wavepackets, reflecting the change that occurred in the correlation of the surface adsorbate molecules over that time. The measured normalised beam polarisation, *P*(*t*)/*P*(*t* = 0), is proportional to the intermediate scattering function (ISF), *I*(Δ*K*,*t*). As this is a scattering technique, the measurements are in the reciprocal space and the ISF is the Fourier transform of the spatial correlation function describing the surface, *G*(*R*,*t*). The ISF hence tells us how quickly the adsorbate molecules “dephase” from their initial positions on picosecond timescales, and the reciprocal length scales are given by 2π/Δ*K*. By considering the variation of the rate of dephasing with temperature and scattering momentum transfer, Δ*K*, we gain a description of the adsorbate motion and can quantify details such as the energy landscape and dissipation rate. More detailed descriptions of the technique are given elsewhere.^1^,^31^

The experiments were performed on a single-crystal Cu(111) sample, mounted in a scattering chamber with a base pressure of 1 × 10^–10^ mbar and cleaned by repeated cycles of Ar^+^ sputtering (800 eV, 10 μA, 20 min at 300 K) and annealing (800 K, 30 s). The surface quality was monitored using the sample’s reflectivity to the helium beam (>20%) and the shape of the helium scattering specular peak. Benzene (>99.9% purity, Aldrich), purified by several freeze–evacuate–thaw cycles, was deposited on the surface by backfilling the chamber and monitoring the drop in the specularly reflected helium beam during adsorption. Using the same method as in our recent study of benzene on Cu(001),^10^ we estimate absolute coverages of 0.03 and 0.1 monolayers (ML) for the low and high coverage measurements, respectively.

## 3. Experimental results

**Fig. 2:**
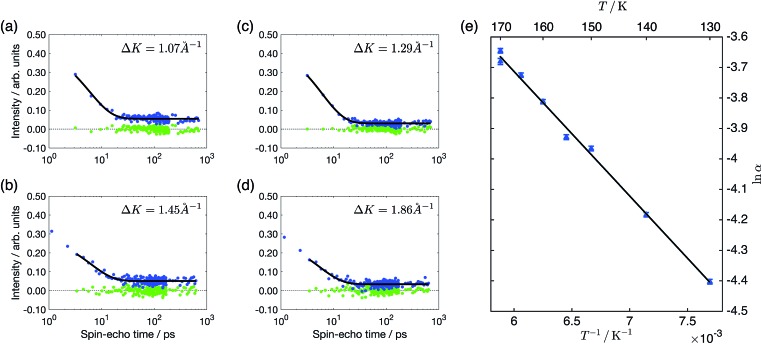
Polarisation measurements as a function of the spin-echo time, *t*, for 0.03 ML (panels a, c) and 0.1 ML (panels b, d) of benzene/Cu(111). The different panels show the measurements at the indicated momentum transfers and a temperature of 170 K. The experimental data are plotted as blue points, a single exponential of the form *f*(*t*) = *a* exp(–*αt*) + *c* from 3 ps is shown as a black line and the residual after subtracting the exponential from the data as green points. The data at times smaller than 3 ps is excluded as it is influenced by the initial fast decay described in the text. Panel (e) shows the temperature dependence of the dephasing rate for a coverage of 0.03 ML at Δ*K* = 0.3 Å^–1^ as an Arrhenius plot. The solid line illustrates the fit to an Arrhenius form, resulting in an effective activation energy of 35 ± 1 meV. All the data were measured along the [112] direction.

The slow decay of the measured polarisation that dominates the ISFs, illustrated in Fig. 2, is indicative of surface diffusion. We can quantify the slow dephasing rate, *α*, by fitting an exponential decay of the form *a*e^–*αt*^ + *c*. However, such a fit of the single exponential decay is only appropriate to quantify motion on a Bravais lattice and, in the case of a non-Bravais lattice, multiple exponential terms are to be expected.^32^ On the Cu(111) surface benzene can sit on a Bravais lattice of adsorption sites in the case where the top site is the energetic minimum, or on a non-Bravais lattice if bridge site adsorption is preferred. The hollow sites form a non-Bravais lattice in the case that the hcp and fcc hollow sites are degenerate in energy, but revert to a Bravais lattice when the hcp and fcc are not degenerate and the adsorbate preferentially adsorbs to one of the two types. By considering whether the measured polarisation shows evidence of multiple decays, we can attribute the likely adsorption sites. Referring to the analytical models detailed by Tuddenham *et al.*,^32^ for bridge or degenerate hollow site adsorption, we would expect the second exponential term to be most apparent at a momentum transfer of *Q*/(2π/*a*) ∼ 0.6 in the [112] direction, corresponding to Δ*K* ∼ 1.5 Å^–1^ on a Cu(111) surface, with the magnitude of this term increasing strongly in the range above 1 Å^–1^. In Fig. 2c, we show the ISF for 0.1 ML coverage at 1.45 Å^–1^, along with a single exponential fit and residuals. It can be seen that there is no evidence of the second decay that would be needed to support a model of bridge or degenerate hollow site adsorption. The presence of a single exponential decay at similar values of momentum transfer is illustrated more widely in panels a–d of Fig. 2, for coverages of both 0.03 ML and 0.1 ML, confirming that there is no evidence of adsorption on a non-Bravais lattice. The experimental data hence suggests adsorption on the top sites or non-degenerate hollow sites.

In Fig. 2e, we present the temperature dependency of the dephasing rate as an Arrhenius plot, at a coverage of 0.03 ML and momentum transfer of 0.3 Å^–1^ in the [112] direction. From the Arrhenius plot, we find an effective activation energy of 35 ± 1 meV, a value approximately one third of that measured for benzene adsorbed on the (001) facet of copper, but very similar to the 41 ± 1 meV measured for five-membered aromatic cyclopentadienyl on the same Cu(111) surface.^9^

In addition to the slow decay that is caused by the diffusive motion of the adsorbate, and which we have analysed above, a typical polarisation measurement also shows an initial rapid drop within the first one or two picoseconds. The much smaller and more rapid decay here is associated with the intracellular motion of the adsorbate molecule when it is localised within a particular adsorption hollow.^32^ In the polarisation curves we present in Fig. 2, it may be noticed that the data at 0.1 ML has two data points taken at smaller times than those shown in the 0.03 ML curves. These two data points are excluded from the subsequent exponential fit illustrated by the black lines, in order to ensure that there is no influence from the initial drop. Once these points are excluded, the slow decay can be considered independently from the fast decay as it occurs on such a different time scale.

## 4. Computational results

As we discussed in the previous section, the experimental data suggest adsorption on top or non-degenerate hollow sites, with a barrier to diffusion of around 35 meV. We now discuss how our computational results point to a global energy minimum for physisorbed benzene on hollow sites, and a weaker local minimum at top sites, which are therefore not likely to be involved in the diffusion process. In order to put the calculations into context, it is useful to summarise here the three main classes of vdW (or dispersion force) correction schemes most commonly employed in surface chemistry. Historically, long-range dispersion-correction schemes have considered differently formulated pairwise atomic potentials that are added to the Kohn–Sham potential energy term. The computational cost of the DFT calculation is therefore essentially unchanged, since the corrective term can be evaluated analytically, and the calculation follows simple approximations derived from the classical electrostatic description of the instantaneous dipole–dipole interactions in London’s formula. The vdW energy term is therefore added at the end of each self-consistent energy minimisation cycle, inside a structural optimisation or transition state search. One can generally classify the semiempirical vdW-corrections according to the transferability and accuracy of the approximations involved in the calculations of the so-called C_6_ coefficients that tune the intensity of the attractive interactions between a given pair of atoms. The simplest approximations (the DFT-D and DFT-D2 schemes developed by Grimme and co-authors^24^,^34^) assume that the C_6_ coefficients are constant during the calculations and independent of the local chemical environment in which the atomic pairs are situated at a given time. More accurate methods (DFT-D3 or TS, for instance) introduce C_6_ coefficients that are dependent on the atomic coordination and local environment (through the atomic polarisabilities and vdW volumes) and therefore offer greater accuracy and predictive power than the older corrections, especially when the corrections are applied to molecular and surface systems in which the same atomic element has several hybridisation states or a varying oxidation state. Finally, the most computationally challenging approach is through the development of vdW-enabled functionals (vdW-DF). These can be described as modified XC functionals that tend to model closely the problematic trend in the asymptotic long-range electron–electron interaction (or lack thereof) in classic LDA and GGA XC-functionals. To do so, they add extra terms in the density derived, for instance, by perturbation theory, Random Phase Approximation (RPA) or local frequency responses (effective plasma frequency).^35^ vdW-DF functionals and DFT-D3 or TS schemes offer a similar level of accuracy for a given benchmark set, with a slight advantage of vdW-DF for some specific combination of XC functional and subsystem. For surface chemistry, the major advantage of the DFT-D3 and TS method in particular is that by adding a negligible amount of additional computing cost to the original DFT calculation, they obtain in most cases exceedingly accurate results, specifically for the reaction and diffusion barriers, although the absolute adsorption energies tend to be overestimated (by about ∼0.2–0.4 eV for small molecules).^36^

In our previous work we benchmarked the performance of the original TS scheme, as implemented in CASTEP, on several aromatic systems: pyrrole/Cu(111),^7^ thiophene/Cu(111)^6^ and benzene/Cu(001).^6^ For pyrrole and thiophene the surface translational/rotational barriers are in very good agreement with the experimental results obtained by HeSE spectroscopy. Taking into account that supramolecular self-assembled systems on metal surfaces have to be modelled with supercells containing hundreds of atoms, the computational-cost advantage of the TS-scheme, when compared to the vdW-DFT functionals, is significant. In this work we have employed the TS and the TSSCS method, which adds self-consistent screening to the original correction scheme, and is thought to be capable of generally improving the performance of the method in adsorbate systems.

**Fig. 3:**
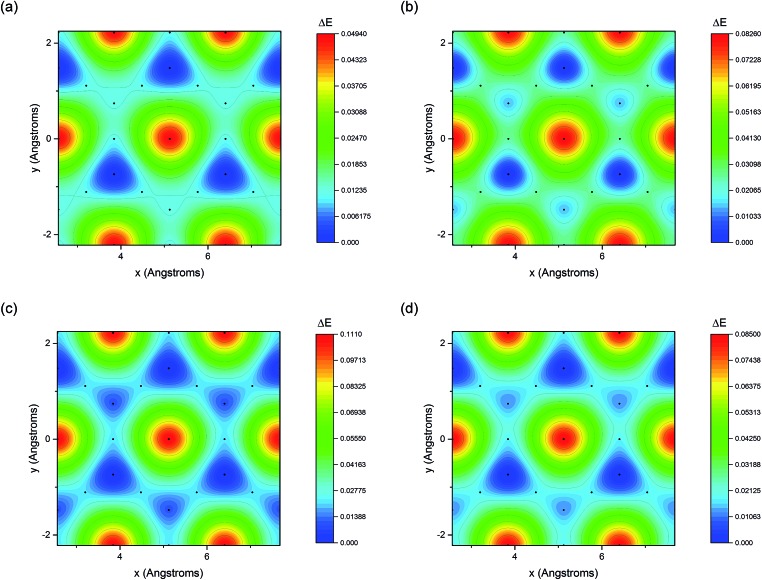
Potential energy surfaces derived from the DFT calculations with (a) G06,^24^ (b) OBS,^25^ (c) TS^26^ and (d) TSSCS^33^ vdW corrections.

The adsorption energy of benzene on the high-symmetry sites on a Cu(111)-(2√3 × 2√3) surface cell is reported in Fig. 3 and Table 1 and compared with selected DFT results from the recent studies of Carter and Rohl.^37^ First of all, it is clear that both the TS and the TSSCS methods agree in indicating the hollow site as the minimum energy adsorption site for benzene. The diffusion barrier is slightly lower for the TSSCS calculations (18 meV instead of 21 meV) and both the TS and TSSCS fall below the experimental results (35 meV), although the difference might be considered to be within reasonable chemical accuracy. The oldest and most primitive correction scheme (G06, ref Grimme *et al.*^24^) also assigns the position of the global minimum to the HCP-R site, but shows a more pronounced difference in the angular dependence of the adsorption energy on TOP sites (0.111 eV). In general, one would not expect Zero Point Energy (ZPE) corrections to be important in the case of a physisorbed aromatic molecule in a completely flat configuration. For this study we calculated the ZPE contributions to the barrier height using DFT with TS vdW corrections. The effect of the ZPE contribution is essentially to shift the minimum energy adsorption site from the HCP to FCC sites, marginally lowering the barrier height between the hollows and BR sites from 21 meV to 17 meV. The HCP and TOP sites are now 86 meV and 180 meV higher in energy than the FCC site. In this paper we will not discuss further the effects of ZPE corrections and the relative accuracy of each pairwise correction scheme for evaluating vibrational frequencies since this will be the subject of future works.

**Table 1:** Energy corrugation, in eV, of the different dispersion energy corrections employed in this study for benzene adsorbed on the high symmetry sites of Cu(111). BR is the bridge site; FCC and HCP the fcc and hcp hollow sites, respectively; TOP is the top site. *I* and *R* indicate the rotational configurations, which differ by a 30 degree rotation around the *z*-axis (Fig. 1). G06 is the vdW correction scheme proposed by Grimme,^24^ OBS is the correction scheme of Ortmann, Bechstedt and Schmidt,^25^ TS and TSSCS are the original Tkatchenko–Scheffler correction scheme and the modified TS scheme with self-consistent screening^26^,^33^

Adsorption site	TS	TSSCS	G06	OBS
BR-I	0.023	0.018	0.018	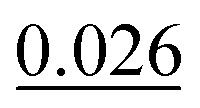
BR-R	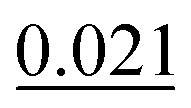	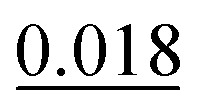	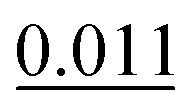	0.045
FCC-I	0.033	0.028	0.023	*0.014*
FCC-R	*0.010*	*0.012*	*0.012*	0.032
HCP-I	0.021	0.014	0.011	**0.000**
HCP-R	**0.000**	**0.000**	**0.000**	0.023
TOP-I	0.125	0.165	0.160	0.082
TOP-R	0.111	0.085	0.049	0.157

**Fig. 4:**
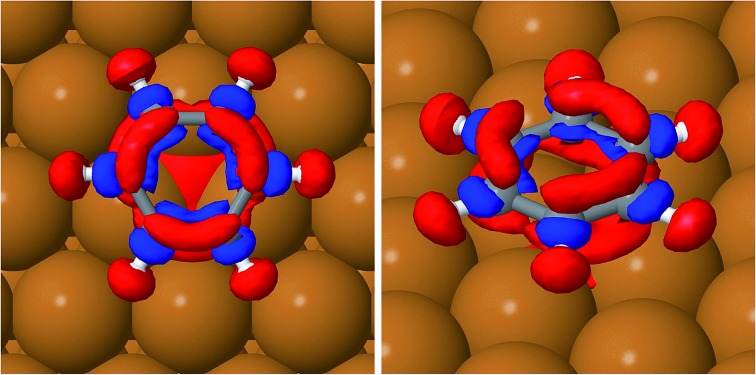
Top view (left) and side view (right) of the charge density difference plots showing charge transfer upon adsorption of benzene on the Cu(111) hcp site in the HCP-R rotational configuration. The red contours indicate an electron density increase by 0.005 electrons per Å^3^; the blue contours indicate an electron density decrease by 0.005 electrons per Å^3^.

Table 2 reports the optimised structure of benzene on the hollow, bridge and top sites. The distance between the molecule and surface at the proposed adsorption site (2.958 Å) is in very good agreement with the reported height of 0.29 nm derived from the work function change measurements.^38^ The accurate position of the molecule on the surface, and therefore the geometrical corrugation of the PES along the *x*, *y* plane,^39^ is the single most important factor in determining the quality of a molecule/surface potential. Our calculations confirm, as previously observed by Carrasco *et al.*^40^ for benzene on transition metal surfaces, that the interaction between benzene and the terraces of the single crystal metal surface is reasonably accurately captured by the original TS scheme and by its related methods. We note that, without vdW corrections, the potential energy curve will become completely unrealistic (see *E*_ads-GGA_ results in Table 2); all the calculated adsorption energies are positive, therefore no binding between benzene and the surface is predicted. The TSSCS corrections, by including the self-consistent screening, also result in good agreement with the experimentally derived adsorption height, while the internal structure of the adsorbate (average C–C bond length, and C–H angle) is essentially identical to the TS values. The moderate downward tilting of the essentially unpolarised C–H bonds does not contribute overall to the stabilisation of the system, by reducing the surface-molecule dipole system, as this is dominated by the binding of the aromatic ring. As described by Witte *et al.*,^41^ the so-called cushion effect is clearly visible in Fig. 4, which shows the electron density-difference plot for benzene adsorbed on the HCP-R site, with a region of electron depletion just above the surface atoms underneath the adsorbate. This quantum chemical effect governs the electronic density of states redistribution between the organic and metal surfaces and it is caused by the Pauli repulsion between the electron density in the layer immediately above the topmost (111) plane and the approaching electron-rich π-system of the aromatic ring. The displaced electron density cloud forms a three-dimensional “cushion” that holds the molecule at approximately 3 Å, causing a total shift of the work-function in the opposite direction to the predictions of the simplistic electrostatic models. A comparison between our results and those reported by Witte *et al.*^41^ shows that the cluster model used in early calculations clearly overestimates the total charge redistribution due to the localised nature of the cluster compared with the periodically repeated surface model employed in the present study. Nevertheless, the understanding of the binding mechanism of benzene on Cu(111) captured by Witte and co-authors within the cushion effect model is essentially correct, especially in predicting the sign of the dipole moment change and consequently the lowering of the work function of the metal surface upon the adsorption of benzene. Witte *et al.* calculated a work function change of –1.08 eV, in excellent quantitative agreement with our calculations (Table 3) at low coverage (1/12 ML), where we found that benzene causes a shift of the work function of 0.99 eV, within 6% of the experimental measurements.^41^ Comparing these results with the work function change induced by the adsorption of Cp on the same surface, as calculated by Sacchi *et al.*,^8^ we notice some similarities, but also a striking difference. We find that the change in the work function does not significantly change when the benzene coverage is increased above 0.11 ML, which is similar to Cp adsorption, where Δ*Φ* changed by only 5% when the coverage was increased from 0.11 to 0.14 ML. Again, we observe some degree of depolarisation in the benzene overlayer when the molecules are compressed beyond a certain critical distance, although it is not clear how far the molecules could be compressed before a critical change in the adsorption angle may occur.^18^

**Table 2:** Adsorption energy with (*E*_ads-vdW_) and without (*E*_ads-GGA_) vdW correction, adsorbate height and average hydrogen–carbon bond angles obtained with PBE + TS corrections for benzene adsorbed on the high symmetry sites of Cu(111)

Surface Site	*E*_ads-vdW_ (eV)	*E*_ads-GGA_ (eV)	Height (Å)	Charge (e)	C–H angle (degrees)
BR-I	–1.027	0.023	2.959	–0.59	–0.663
BR-R	–1.029	0.038	2.972	–0.56	–0.183
FCC-I	–1.018	0.009	2.955	–0.59	–0.777
FCC-R	–1.040	0.031	2.967	–0.60	–0.211
HCP-I	–1.030	0.016	2.960	–0.59	–0.380
HCP-R	–1.050	0.003	2.958	–0.58	–0.313
TOP-I	–0.926	0.098	2.971	–0.48	–1.444
TOP-R	–0.940	0.093	3.174	–0.60	–1.437

**Table 3:** Calculated surface dipole moment, Δ*μ* (D) and the work function change Δ*Φ* (eV) for benzene adsorbed on Cu(111) at increasing coverage (ML)

Coverage (ML)	Δ*μ* (D)	Δ*Φ* (eV)
0.08	1.8	–0.99
0.11	2.3	–1.70
0.14	1.7	–1.62

**Fig. 5:**
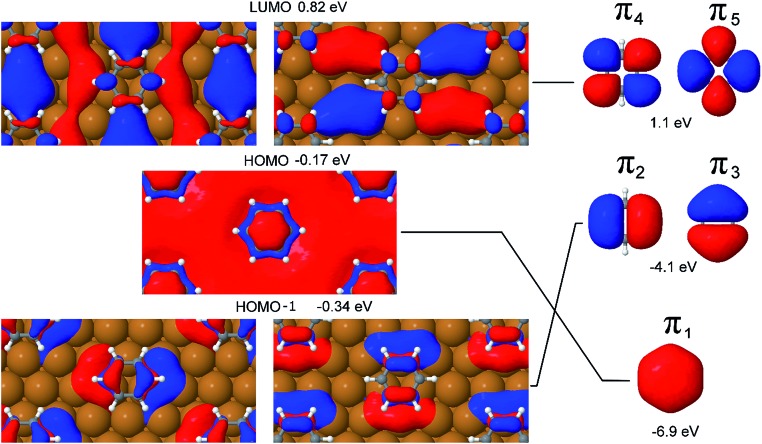
Left, the LUMO (0.82 eV), HOMO (–0.17 eV) and HOMO–1 (–0.34 eV) Kohn–Sham orbitals (KS-MOs) at the gamma point for benzene adsorbed on a hollow hcp site. Right, from top to bottom, the symmetry related pi orbitals for isolated benzene. The KS-MOs of the surface are joined by solid lines with the symmetry equivalent pi orbitals of benzene. The π_3_, π_2_ and π_1_ orbitals are much lower in energy than the correspondent surface mixed-character KS-MOs and are represented beside the latter for the purpose of easy visualisation.

The position of the lowest energy structure and the measured barrier for jump diffusion are entirely consistent with the previous measurements on Cp/Cu(111). In principle, one would not expect such a similarity in the behaviour of these two molecules, given the different symmetry groups (*D*_6h_ and *D*_5h_). It is observed that the substrate reduces the symmetry of both molecules and the extent of charge backtransfer in Cp more than compensates for the larger dispersion interaction between the six-membered ring and surface. The slightly higher barrier for the jump diffusion of cyclopentadienyl on the same surface can be partly rationalised by an increase in the ionic binding, combined with the reduced symmetry compatibility between the pentagonal aromatics and hexagonal substrate. The local adsorption site (hollow hcp) has a reduced three-fold symmetry with respect to the six-fold rotational symmetry of the clean surface and the reduced overlap between the d_*z*_ orbitals of the three Cu atoms with the π-system of benzene is clearly visible in the density difference plot of Fig. 4. The molecular orbital mismatch between the substrate and molecule (Fig. 4 and 5) reduces the height of the rotational barrier compared to what we observed for benzene on Cu(001), where the experimental and theoretical activation energy are a factor of 3 and 35 higher than for the Cu(111) surface, respectively. The much greater energetic corrugation of the Cu(100) surface is therefore captured by the vdW-corrected DFT simulations, but with a clear DFT overestimation of the barrier height for the jump diffusion (hollow to hollow site) for Cu(100). It is also worth discussing the charge transfer between the substrate and adsorbate in different locations on the PES. For smaller molecules, the extent of donation and backdonation determines the intensity of the binding energy. However, for benzene on Cu(111), the charge transfer from the surface to the molecule does not seem to be correlated to either the distance between the molecule and the surface, or the adsorption energy. Still, it is worth noting that the highest energy local minimum (TOP-I) does show the lowest degree of electron backdonation (–0.4 e) from the substrate.

It is evident from Fig. 5, which shows the Kohn–Sham orbitals (KS-MOs) at the gamma point for benzene adsorbed on a hollow hcp site, that the extremely low chemical interaction between the Cu(111) surface and the adsorbate does not translate into a negligible mixing between the surface and molecular orbitals. In fact, if we concentrate our attention on the frontier orbitals of the system (a selection of these, the HOMO, HOMO–1 and LUMO are represented in Fig. 5), the high degree of mixing between the surface state and molecular π states is immediately visible in the Kohn–Sham orbital representations of these states. For instance, in the HOMO–1 the lower lobes of the π_2_ and π_3_ orbitals extend to the copper atoms surrounding the hollow site, while the character of the π_4_ and π_5_ orbitals in the LUMO is completely mixed with the surface states connecting adjacent molecules. Furthermore, the calculations show that the π_1_ and (π_2_, π_3_) orbitals are blue shifted in energy by 6.7 eV and 3.8 eV respectively to become part of the HOMO and HOMO–1 bands.

Comparing the results of the calculations with those reported by Carter *et al.*, we can observe a substantial agreement between our data and the results of optB88-vdW, revPBE (vdW-DF) and rPW86 (vdW-DF2). In particular, the molecule-surface height is very similar to that which Carter *et al.* reported for the vdW-DF (optB88-vdW) calculations (2.93 Å) and the barrier height is also entirely consistent with the results of the vdW-DF calculations (in the 0–30 meV range).^37^ The binding energy calculated by the TS and TSSCS methods overestimates the experimental adsorption energy (by about 0.4 eV) in the same way as less accurate correction schemes OBS and G06. The barrier for diffusing from HCP over the BR site is about 20 meV, with FCC lying around 10 meV higher than HCP. The energetics of the diffusion pathway do not seem to change significantly with the choice of the vdW corrections with TS, TSSCS, G06 and OBS schemes resulting in barriers in the 11–26 meV range.

All four schemes also result in energetic differences between the HCP and FCC hollows of only 10–14 meV. The experimental data supports adsorption to the non-degenerate hollow sites as the ISFs did not show the additional decay that would be expected in the degenerate case. However, there is a further subtlety. The measurements were carried out at 170 K, corresponding to a thermal energy of 15 meV. If the energetic differences were indeed as small as 10–14 meV, we would expect to have to consider the contribution from jumps from the FCC sites, which would then be reasonably occupied. The ratio of jump rates from the HCP to FCC and FCC to HCP sites is given by *λ* = exp(Δ*E*/*k*_B_*T*) where Δ*E* is the energy difference of the two sites. For the calculated energies, *λ* is in the range of 2–3. Tuddenham *et al.* illustrate that for *λ* = 2 (equivalent to a 10 meV difference), a second exponential term would still be expected, but would become most apparent at slightly higher momentum transfers (around 1.8 Å^–1^), an effect that is not seen in Fig. 2d. Due to the exponential nature of *λ*, the strength of the second exponential term rapidly falls away in magnitude as Δ*E* increases, which suggests that the calculations are probably underestimating the difference between the two sites to a similar degree to the diffusion barrier, where the calculations are 10–25 meV lower than the experiment. A FCC–HCP difference in energy of just 25 meV would be indiscernible within the present experimental data from the greater difference.

The small energetic difference between the *I* and *R* rotational states (11–23 meV on the energetically preferred site) would lead to the occurrence of both rotational states at the temperatures of the experiment. However, if we consider the energies presented in Table 2, although these states will be present frequently, they do not affect the rate-limiting barrier, which is from HCP-R to BR-R for TS and TSSCS, HCP-R to FCC-R for G06 and HCP-I to BR-I for OBS. The population of the rotated states would be expected to affect the pre-exponential factor for diffusion, rather than the Arrhenius activation energy that was considered in this work. A typical molecule’s trajectory across the surface would be expected to involve rotation as well as translation, but without the degree of coupling between the two that might be described in terms of quasi-static steering^10^ where the molecule must rotate to pass over the rate-limiting barrier.^42^

## 5. Conclusions

Benzene adsorption on Cu(111) has been established as a benchmark system for exploring the 2D dynamics of self-assembled aromatics and conjugated molecules. In this work we have shown that benzene adsorbs on the hollow sites of the Cu(111) surface and undergoes jump-diffusion with an extremely low barrier height (35 ± 1 meV, according to the HeSE results, compared with 12–26 meV for the DFT calculations with different vdW correction schemes). The potential energy landscape for this system is well characterised by the combination of a GGA XC-functional (PBE) and vdW corrections with an accuracy that is comparable with that of modern DFT-DF functionals. The HeSE results clearly exclude that benzene might adsorb, as previously suggested, on bridge sites and the DFT calculations help to understand that atop sites, although compatible with the jump-diffusion mechanism, are not energetically the most favourable. Rotation around the molecular central C_6_ axis is also an activated process that shows a barrier height (12–23 meV) that is essentially identical to that of the jump-diffusion. By analysing the electron density redistribution upon adsorption and the change in the ordering and energetics of the molecular orbitals interacting with the surface bands, we rationalise the observed small, but finite, barriers for diffusion in terms of the symmetry mismatch between the *D*_6h_ symmetry of the MOs and the three-fold symmetry of the charge backdonated to the molecule.
